# Relationship of thyroid function with intracranial arterial stenosis and carotid atheromatous plaques in ischemic stroke patients with euthyroidism

**DOI:** 10.18632/oncotarget.14883

**Published:** 2017-01-28

**Authors:** Junfeng Liu, Xiaoyang Cui, Deren Wang, Simiao Wu, Yao Xiong, Shihong Zhang, Bo Wu, Ming Liu

**Affiliations:** ^1^ Department of Neurology, Stroke Clinical Research Unit, West China Hospital, Sichuan University, Chengdu, P.R. China; ^2^ Department of Respiratory and Critical Care Medicine, China-Japan Friendship Hospital, Beijing, P.R. China

**Keywords:** free triiodothyronine, free thyroxine, thyroid-stimulating hormone, intracranial artery stenosis, carotid atheromatous plaques

## Abstract

This study aimed to help clarify the possible relationships of thyroid function with intracranial arterial stenosis or carotid atheromatous plaques in ischemic stroke patients with euthyroidism. We retrospectively reviewed the medical records of a consecutive series of ischemic stroke patients prospectively entered into the Chengdu Stroke Registry between February 2010 and March2012. We performed univariate and multivariate analysis to assess possible relationships of thyroid function with intracranial artery stenosis or carotid atheromatous plaques. Of the 172 patients analyzed (42 women; 61.7 ± 14.0 years old), 62 (32.0%) had carotid atheromatous plaques, and 81 (47.1%) had intracranial artery stenosis. Free thyroxine levels were lower in patients with carotid atheromatous plaques than in patients without plaques (15.80±2.09 vs. 16.92±2.69, *P* = 0.005). After adjusting for age, gender, hyperlipidemia, and previous smoking, free thyroxine levels were independently associated with carotid atheromatous plaques (OR 0.73, 95% CI 0.54-0.99, *P* = 0.04). In contrast, thyroid function indicators showed no associations with intracranial arterial stenosis. In conclusion, low free thyroxine levels were independently associated with carotid atheromatous plaques in ischemic stroke patients with euthyroidism, but thyroid function indicators were not associated with intracranial artery stenosis.

## INTRODUCTION

Stroke is the second most common cause of death after cancer and a leading cause of adult disability worldwide [1]. Approximately 85% of strokes are ischemic strokes [2]. Stroke incidence appears to be increasing, and the associated economic costs are staggering [3]. Population-or hospital-based prospective studies and large randomized trials have identified carotid plaques [4–6]and major intracranial arterial stenosis[7–9 ]as major causes of stroke, especially in Asians, Blacks, and Hispanics. Thus, identifying easily screenable factors that may correlate with the presence or severity of carotid plaques or major intracranial arterial stenosis may help identify individuals at risk of stroke.

Thyroid dysfunction, including overt hyper-or hypothyroidism as well as subclinical thyroid disease, has been associated with cardiovascular disease [10], cerebrovascular risk [11, 12], atherosclerosis [13, 14] and thickening of the carotid artery intima media [15, 16]. In addition, thyroid function at the low end of the normal range (still within euthyroid limits) has been associated with increased levels of inflammatory markers [17], thickening of the carotid intima media [18–21], stenosis of the internal carotid artery [22], and ulceration of carotid artery plaques [23]. These associations suggest that thyroid function may be associated with intracranial arterial stenosis and/or carotid atheromatous plaques in ischemic stroke patients, even in patients with euthyroidism. However, we are unaware of published work on this question.

Therefore the present study investigated the impact of thyroid function on intracranial arterial stenosis and carotid atheromatous plaques in ischemic stroke patients with euthyroidism.

## RESULTS

**Figure 1:**
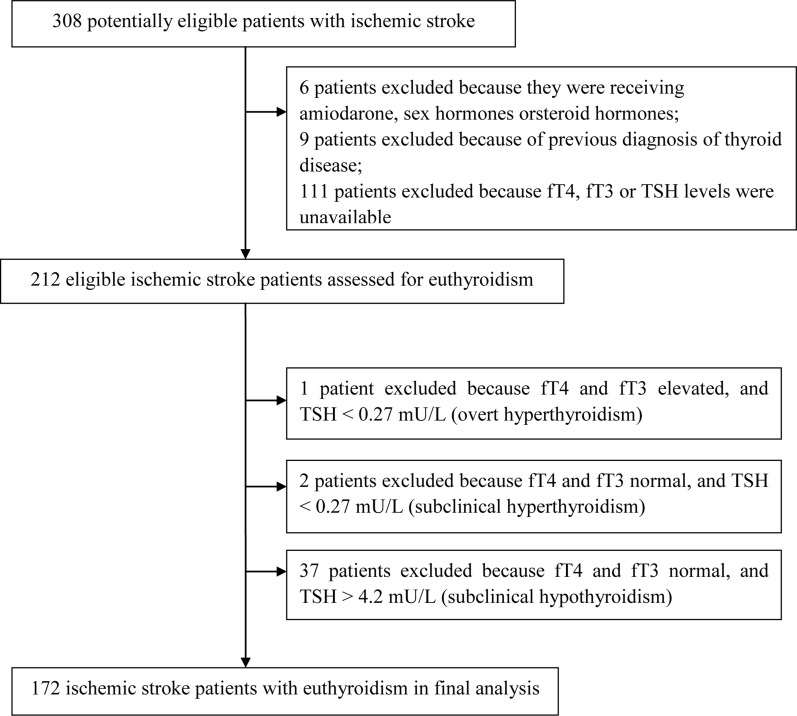
Study population and algorithm

During the study period, 308 patients suffering first-ever or recurrent ischemic stroke were enrolled in the Chengdu Stroke Registry. After excluding patients who met the exclusion criteria, 212 were analyzed closely for euthyroidism (Figure 1). Patients were excluded from this group because their fT3 and fT4 levels were in the normal range and TSH levels>4.2 mU/L, corresponding to subclinical hypothyroidism (n=37); because their fT3 and fT4 levels were in the normal range and TSH levels<0.27mU/L, corresponding to subclinical hyperthyroidism (n=2); or because their fT3 and fT4 levels were elevated and TSH levels<0.27 mU/L, corresponding to overt hyperthyroidism (n = 1). The remaining 172 patients with euthyroidism were included in the final analysis.

Age did not differ significantly between patients in the final analysis and those who were excluded (61.7 ± 14.0 *vs* 63.2± 14.3 yr, *P*=0.339). The proportion of women was significantly higher among excluded patients than included patients (43.4 *vs* 24.4%, *P*<0.001).

Among the 172 patients with euthyroidism, 81 (47.1%) had intracranial artery stenosis, and 62(32.0%) had atheromatous plaques in carotid arteries. Baseline characteristics of patients stratified according to the presence or absence of carotid atheromatous plaques or intracranial artery stenosis are presented in Table 1. Patients with carotid atheromatous plaques were older than those without plaques and more likely to be male, to have a smoking history, and to have hyperlipidemia. In contrast, patients with or without intracranial artery stenosis were similar in terms of age, gender, risk factors of stroke, as well as smoking and drinking status.

**Table 1:** Baseline characteristics of ischemic stroke patients stratified by carotid atheromatous plaques or intracranial artery stenosis

	Plaques (*n*= 62)		No plaques (*n*= 110)	*P*	Stenosis (*n*#x003D; 81)	No stenosis (*n* = 91)	*P*
**Age, yr**		64.82±13.48	59.91±14.04	**0.03**	61.16±14.56	62.14±13.56	0.65
**Male**		54(87.1%)	76(69.1%)	**0.008**	61(75.3%)	69(75.8%)	0.94
**Hypertension**		32(51.6%)	63(57.3%)	0.47	46(56.8%)	49(53.8%)	0.70
**Diabetes mellitus**		14(22.6%)	16(14.5%)	0.18	18(22.2%)	12(13.2%)	0.12
**Hyperlipidemia**		10(16.1%)	6(5.5%)	**0.02**	9(11.1%)	7(7.7%)	0.44
**Atrial fibrillation**		3(4.8%)	9(8.2%)	0.41	9(11.1%)	3(3.3%)	0.05
**Coronary heart disease**		7(11.3%)	8(7.3%)	0.37	5(6.2%)	10(11.0%)	0.26
**Previous stroke**		8(12.9%)	15(13.6%)	0.89	14(17.3%)	9(9.9%)	0.16
**Smoking consumption**		32(51.6%)	37(33.6%)	**0.02**	32(39.5%)	37(40.7%)	0.88
**Alcohol consumption**		17(27.4%)	26(23.6%)	0.58	21(25.9%)	22(24.2%)	0.79
**TSH, mU/L**		1.79±0.88	1.91±1.01	0.43	1.77±0.99	1.96±0.93	0.20
**FT4, pmol/L**		15.80±2.09	16.92±2.69	**0.005**	16.70±2.71	16.35±2.38	0.37
**FT3, pmol/L**		4.09±0.77	3.99±0.85	0.48	3.99±0.80	4.06±0.85	0.58

Levels of fT4 were significantly lower in patients with carotid atheromatous plaques than in patients without plaques, while these levels were similar between patients with or without intracranial artery stenosis (Table 1). In the multivariate model (Table 2), fT4 levels were still independently associated with carotid atheromatous plaques. Levels of TSH and fT3 were similar between patients with or without carotid atheromatous plaques, and between patients with or without intracranial artery stenosis.

**Table 2:** Multivariate analysis to identify association of thyroid function (treated in continuous parameters) with carotid atheromatous plaques

	OR	95%CI	*P*
**Age, yr**	1.03	1.01-1.06	0.02
**Male**	2.02	0.76-5.35	0.16
**Smoking consumption**	1.58	0.74-3.37	0.23
**Hyperlipidemia**	2.56	0.82-7.99	0.11
**TSH, mU/L**	0.90	0.62-1.32	0.80
**FT4, pmol/L**	**0.82**	**0.70-0.96**	**0.01**
**FT3, pmol/L**	1.38	0.89-2.15	0.16

The raw (continuous) levels of TSH, fT4 and fT3 were then converted into quartiles (Table 3) in order to perform logistic regression to identify possible associations of thyroid function with carotid atheromatous plaques or intracranial artery stenosis. Only one significant association was found: patients in lower fT4 quartiles were at significantly higher risk of carotid atheromatous plaques than patients in the highest quartile. This relationship persisted even after adjusting for age, gender, hyperlipidemia, and smoking consumption (OR 0.73, 95% CI 0.54-0.99, *P*= 0.04, Table 4).

**Table 3:** Univariate analysis to identify association of thyroid function (treated categorically in quartiles) with carotid atheromatous plaques or intracranial artery stenosis

	Quartile 1	Quartile 2	Quartile 3	Quartile 4
**TSH, mU/L**				
**Median (range)**	0.72(0.29-1.07)	1.38(1.13-1.75)	2.20(1.80-2.38)	3.07(2.40-4.20)
**Patients with plaques, n/N (%)**	18/43(41.9)	15/43(34.9)	16/43(37.2)	13/43(30.2)
**OR (95% CI)**	1.66(0.68-4.04)	1.24(0.50-3.05)	1.37(0.56-3.36)	Ref
**Patients with stenosis, n/N (%)**	25/43(58.1)	20/43(46.5)	14/43(32.6)	22/43(51.2)
**OR (95% CI)**	1.33(0.57-3.11)	0.83(0.36-1.94)	0.46(0.19-1.11)	Ref
**FT4, pmol /L**				
**Median (range)**	14.00(11.24-14.92)	15.43(14.94-16.06)	17.00(16.09-17.99)	19.32(18.02-26.92)
**Patients with plaques, n/N (%)**	21/43(48.8)	18/43(41.9)	12/43(27.9)	11/43(25.6)
**OR (95% CI)**	**2.78(1.12-6.89)**	2.10(0.84-5.23)	1.13(0.43-2.93)	Ref
**Patients with stenosis, n/N (%)**	19/43(44.2)	18/43(41.9)	21/43(48.8)	23/43(53.5)
**OR (95% CI)**	0.69(0.29-1.61)	0.63(0.27-1.47)	0.83(0.36-1.94)	Ref
**FT3, pmol /L**				
**Median (range)**	3.14(1.67-3.46)	3.90(3.48-4.08)	4.27(4.09-4.57)	5.00(4.58-6.52)
**Patients with plaques, n/N (%)**	17/44(38.6)	13/44(29.5)	17/42(40.5)	15/42(35.7)
**OR (95% CI)**	1.13(0.47-2.72)	0.76(0.31-1.87)	1.22(0.51-2.96)	Ref
**Patients with stenosis, n/N (%)**	21/44(47.7)	19/44(43.2)	21/42(50.0)	20/42(47.6)
**OR (95% CI)**	1.00(0.43-2.34)	0.84(0.36-1.96)	1.10(0.47-2.59)	Ref

Ref, reference; CI, confidence interval

**Table 4:** Logistic regression to identify association of thyroid function with carotid atheromatous plaques

Indicator (in quartiles)	OR	95% CI	*P*
**TSH**	0.91	0.68-1.23	0.55
**FT4**	**0.73**	**0.54-0.99**	**0.04**
**FT3**	1.00	0.74-1.35	0.99

Data were adjusted for age, gender, hyperlipidemia, and smoking consumption.

## DISCUSSION

The present study suggests that low fT4 levels are independently associated with the presence of carotid atheromatous plaques in ischemic stroke patients with euthyroidism. Given that carotid plaques are associated with subsequent ischemic stroke [4–6], our data support the possibility that low-normal thyroid function can increase cerebrovascular risk, suggesting the need to improve thyroid screening and to redefine optimal ranges of thyroid hormone serum concentrations.

Our results are consistent with previous studies showing associations between low fT4 levels and elevated levels of inflammatory markers [17], mitral annular calcification [24], and thickening of the carotid intima media [18–21] in stroke patients with euthyroidism. Since our findings were obtained with a relatively large and inclusive sample of ischemic stroke patients, we strengthen the case for targeted screening of ischemic stroke patients with low-normal thyroid function for carotid atheromatous plaques.

The observed association between low fT4 levels and prevalence of carotid atheromatous plaques can be explained by several possible mechanisms. Low fT4 levels may promote atherosclerosis indirectly by increasing lipid levels, insulin resistance, and hypertension, as well as by impairing endothelial function [25–28]. In addition, thyroid hormones have a direct vasodilatory effect, acting mainly on vascular smooth muscle cells [29]. Therefore low fT4 levels may facilitate the formation and/or growth of carotid atheromatous plaques.

Possible factors may help explain why fT3 was not associated with plaques in our patients. These factors include the short half-life of fT3, the possibility that serum concentrations of fT3 do not accurately reflect concentrations in tissues, and the greater sensitivity of fT3 than fT4 to hyperthyroidism, which may not be associated with carotid artery plaques. Further work should verify and extend our findings about fT3 in ischemic stroke patients.

We observed no significant association between any of the three thyroid hormones and intracranial artery stenosis in our patients. These results are consistent with studies on young Chinese stroke patients with euthyroidism [30] and Chinese stroke patients with hyperthyroidism [31]. Those studies also found no association of TSH, fT4, or fT3 levels with intracranial artery stenosis. They did, however, find such stenosis to be associated with elevated thyroid autoantibodies. This association should be confirmed and extended in future work. The different association of thyroid indicators with carotid atheromatous plaques or intracranial artery stenosis may reflect that intra- and extracranial vessels differ in anatomic location as well as in atherosclerotic pathophysiology [32].

Indeed, intracranial arterial stenosis differs from carotid atheromatous plaques in epidemiology and risk factors. Stenosis occurred in 47.1% of our patients, while carotid plaques occurred in 32.0%, similar to previous studies in Chinese [33] and other Asians [32, 34–37], suggesting that intracranial arterial stenosis is more prevalent among Asians. Moreover, we found carotid atheromatous plaques to correlate positively with age, gender, hypercholesterolemia and previous smoking, consistent with previous work [38]; such correlations were not observed for stenosis.

This study has some limitations. First, it was a retrospective, cross-sectional, hospital-based study, which means that cause-and-effect relationships between fT4 levels and carotid atheromatous plaques cannot be explored. Second, the patients we excluded were more likely to be female; this may have confounded our results, since the function of the hypothalamic-pituitary-thyroid (HPT) axis shows subtle sex differences [39], and men are more likely than women to have atherosclerosis [40]. To reduce the potential effects of such bias, we adjusted for age, gender, hyperlipidemia, and previous smoking in the multivariate analysis, which still indicated a significant relationship between low fT4 levels and carotid atheromatous plaques. Third, we measured fT4, fT3 and TSH levels only once and we did not measure thyroid autoantibody levels. This may have led to misdiagnosis of thyroid disease in some cases, since levels of thyroid hormones, especially TSH, can vary in patients with acute ischemic stroke [38, 41]. To reduce the potential confounding effects of non-thyroidal illness on thyroid function, we excluded patients who had abnormal TSH values or who were taking thyroid medication. Longitudinal studies involving larger samples are required to verify our findings on the influence of thyroid function on carotid atheromatous plaques and intracranial arterial stenosis.

## CONCLUSIONS

Low fT4 levels were independently associated with carotid atheromatous plaques in a consecutive sample of Chinese ischemic stroke patients with euthyroidism. In contrast, no association was found between any of the three thyroid hormones tested and intracranial artery stenosis.

## MATERIALS AND METHODS

This study involved retrospective analysis of medical records that had been entered prospectively into the Chengdu Stroke Registry, a database on consecutive ischemic stroke patients treated at West China Hospital since 2005. The study protocol was approved by the Scientific Research Department of West China Hospital. Written informed consent was obtained from participants or their guardians.

The present study involved the set of Registry patients admitted to the neurology department of West China Hospital, Sichuan University between February 2010 and March 2012 [33]. Patients were diagnosed with first-ever or recurrent stroke according to World Health Organization(WHO) criteria [42]. Diagnosis was confirmed using computed tomography (CT) or magnetic resonance imaging (MRI). All patients underwent a thorough stroke workup [33, 43] in accordance with international standards, including neurological examination, brain CT or MRI (T1, T2, and angiography), CT angiography of the aortic arch and carotid arteries, 12-lead electrocardiography, and blood biochemistry.

Patients were excluded from the study if (1)they had previously been prescribed amiodarone, sex hormones or steroid hormones; (2)they had previously been diagnosed with thyroid disease; or (3)their levels of free triiodothyronine (fT3), free thyroxine (fT4), or thyroid-stimulating hormone(TSH) on the morning after hospital admission were unavailable.

Data were collected at the time of patient assessment using a standardized form. We collected patient demographic characteristics (age and gender) and stroke risk factors (hyperlipidemia, hypertension, diabetes mellitus, smoking consumption, and alcohol consumption). Stroke severity on admission was assessed using the National Institutes of Health Stroke Scale (NIHSS) score [44].

Blood samples were collected from each participant on the morning following admission. Levels of fT3, fT4 and TSH were measured using commercial radioimmunoassay kits and compared to the following normal reference ranges: fT3, 3.6-7.5 pmol/L; fT4, 12-22pmol/L; and TSH, 0.27-4.2 mU/L. During analysis, patients were assigned to quartiles depending on their levels of each hormone. The ranges for fT3 quartiles (pmol/L) were as follows: Quartile1, ≤3.46; Quartile 2, 3.46-4.08; Quartile 3, 4.08-4.57; and Quartile 4, ≥4.57. The ranges for fT4 quartiles (pmol/L) were as follows: Quartile1, ≤14.93; Quartile 2, 14.93-16.08; Quartile 3, 16.08-18.01; and Quartile 4, ≥18.01. The ranges for TSH quartiles (mU/L) were as follows: Quartile1, ≤1.09; Quartile 2, 1.09-1.78; Quartile 3, 1.78-2.40; and Quartile 4, ≥2.40.

Diagnosis of intracranial artery stenosis [45–46] was based on ≥50% reduction in the luminal diameter of at least one major intracranial artery, defined as the intracranial internal carotid artery, anterior cerebral artery, middle cerebral artery, posterior cerebral artery, posterior inferior cerebellar artery, intracranial vertebral artery, or basilar artery. Luminal diameter was determined by CT angiograms, on which arteries usually appear homogeneously full of contrast material, and arterial walls appear as a sharp boundary [33]. Diagnosis of a carotid atheromatous plaque [33] was based on the absence of contrast material adjacent to the arterial wall. Two neurologists blinded to clinical information independently evaluated CT angiograms. A third neurologist arbitrated in case of disagreement, and a consensus decision was reached. The inter-class correlation coefficient (ICC) for the two reviewers was 0.876 (95% CI 0.720–0.816), indicating very good inter-observer agreement.

Data for continuous variables were reported as mean ± standard deviation (SD), and data for categorical variables as absolute or relative frequencies. Intergroup differences in categorical variables were assessed for significance using the chi-squared or Fisher's exact tests, while differences in continuous variables were assessed using one-way analysis of variance (ANOVA) or the Mann–Whitney U test. Patients were first compared based on the raw (continuous) values of thyroid hormones. Then they were classified into quartiles for logistic regression to identify associations of thyroid function with intracranial artery stenosis or carotid atheromatous plaques. Variables with *P*< 0.1 in univariate analysis were included in multivariate logistic regression. All statistical analysis was performed using SPSS 20.0 (IBM, Chicago, IL, USA). Two-sided *P*<0.05 was considered to be statistically significant.
